# The role of chamaejasmine in cellular apoptosis and autophagy in MG-63 cells

**DOI:** 10.1042/BSR20181707

**Published:** 2019-01-15

**Authors:** Dawei Yang, Hao Zhang, Jianjun Wu, Ruishuang Ma, Zongyu Li, Kunzheng Wang, Fan Yang

**Affiliations:** 1 Department of Orthopedics, The Fourth Affiliated Hospital of Harbin Medical University, Harbin, China; 2 Department of Orthopedic Surgery, The Fifth Hospital of Harbin, Harbin, China; 3 Department of Cardiology, The Second Affiliated Hospital of Harbin Medical University, Harbin, China; 4 The Department of Internal Medical Oncology, Harbin Medical University Cancer Hospital, Harbin, China; 5 Department of Orthopedics, The Second Affiliated Hospital of Xi’an Jiaotong University, Xi’an, China; 6 Key Laboratories of Education Ministry for Myocardial Ischemia Mechanism and Treatment, Harbin, Province Heilongjiang, China

**Keywords:** autophagy, apoptosis, AMPK/mTOR, chamaejasmine, osteosarcoma

## Abstract

Background: Osteosarcoma (OS) is the most common malignant neoplasm in children and adolescents with a very high propensity for local invasion and poor response to current therapy. Anti-cancer effect of chamaejasmine is newly discovered from Stellera chamaejasmine L. Our study focuses on investigating the effect of chamaejasmine on the cellular apoptosis, proliferation, autophagy, and the underlying mechanisms in MG-63.

Methods: Our study investigated the concentration of chamaejasmine in MG-63 cells by MTT and verified that chamaejasmine inhibited cell invasion by transwell. We also used Hoechst staining as well as apoptotic associated-proteins in MG-63 cells. Meanwhile, we also detected the lysophagesome and autophagsome by Lysotracker. Adenosine 5′-monophosphate (AMP)-activated protein kinase (AMPK) knockdown was performed with siRNA.

Results: Our results show that chamaejasmine exerts cellular growth inhibition, pro-apoptotic and pro-autophagic effect via activating AMPK in MG-63 cells. Furthermore, chamaejasmine significantly increases autophagic cell via the inhibition of mammalian target of rapamycin (mTOR) and activation of AMPK signaling pathways. Administrated with chamaejasmine also induces reactive oxygen species (ROS) generation, indicating cross-talking between these two primary modes of programmed cell death.

Conclusion: Our results show that chamaejasmine promotes apoptosis and autophagy by activating AMPK/mTOR signaling pathways with involvement of ROS in MG-63 cells. Chamaejasmine is a promising anti-cancer agent in OS treatment, and further studies are needed to confirm its efficacy and safety *in vivo* or other cancer cells.

## Introduction

Osteosarcoma (OS) is the most common type of primary malignant bone tumor, ordinarily manifesting as malignant mesenchymal cells producing osteoid or immature bone [1]. The improvement of medical techniques has led to an obvious increase of 5-year survival rate of OS [2]. Although a large number of chemotherapeutic agents are currently in use, most of patients need limb amputation and undergo lung metastasis [3]. Until now, the pathogenesis and molecular mechanisms of osteosarcoma are still unknown. Therefore, novel strategies are essential to develop more effective therapies for OS treatment.

Many plants have anti-tumor pharmacological effects, and about two-thirds of current anti-cancer drugs such as vinblastine, topotecan, etoposide, and paclitaxel are originally plant-derived compounds [4,5]. ‘Lang-Du’(Chamaejasmine), as a traditional Chinese herbal, has been reported that chamaejasmine has an anti-tumor activity [6]. It is one of the major bioactive ingredients of Stellera chamaejasmine L [7]. However, to the best of knowledge, there has been little research on chamaejasmine and/or elucidating the anti-tumor molecular mechanisms of actions in osteosarcoma cells.

Apoptosis is an important self-stabilizing mechanism for multicellular organisms [8], which is characterized by particular morphological changes, including plasma membrane bleb, cell shrinkage and DNA fragmentation [9]. It is reported that complex interaction of molecular events results in the activation of the apoptotic cascade [10]. Studies reveal that reactive oxygen species (ROS) generation and disruption of the mitochondrial membrane potential contribute to drug-induced apoptosis [11,12]. In normal cells, autophagy is essential for the maintenance of cellular energy and is responsible for the regulation of organelle turnover [13]. In response to various cellular stresses, autophagy is up-regulated in order to prevent the accumulation of misfolded or damaged proteins and organelles [14–17]. As immortal cells, the occurrence of tumor cells is not only with the abnormal cell biogenesis, but also with the abnormalities of apoptosis. It has been demonstrated that spontaneous apoptosis in malignant tumors may have therapeutic effects on tumors [18]. Accumulating evidence has linked the apoptosis to autophagy [19]. Thus, apoptosis or/and autophagy are interesting mechanisms to induce the death of cancer cells. Until now, the role of apoptosis in cancer therapy is still controversial.

Adenosine 5′-monophosphate (AMP)-activated protein kinase (AMPK) is an evolutionarily conserved sensor of cellular energy status that is activated by pathological stresses such as oxidative and cellular stresses that deplete ATP and increase AMP [20–25]. Cell growth, proliferation, differentiation, and cell cycle regulation could be controlled by mammalian Target of Rapamycin (mTOR). It is reported that activation of mTOR suppresses autophagy, and the inhibition of mTOR activity promotes it [26–29]. As the mechanism of action, AMPK activation regulates tumor cell growth through the inhibition of mTOR pathway, which is recognized to be associated with translation initiation and protein synthesis [30,31].

Our study aimed to demonstrate the anti-cancer effects of chamaejasmine against MG-63 cells by investigating its effects on apoptosis increasing, inhibition of cell invasion, and autophagy induction. To the best of our knowledge, the present constitutes the first report on this plant species in OS as its pro-autophagic effect.

## Methods and materials

### Cell lines and chemicals

Osteosarcoma cell lines MG-63/U2Os/Saos-2/KHOs were obtained from the Chinese Academy of Sciences (Shanghai, China) and cultured in DMEM complemented with 10% FBS and antibiotics in 37°C humidified atmosphere with 95% O_2_ and 5% CO_2_. Chamaejasmine (purity ≥98%) was obtained from the Zhongbiao Biochemistry Inc. (Wuhan, China). Compound C (SML2017-5mg), Bafilomycin A1 (02911643), NAC (A9165-5g) and 3-MA (M9281-100MG) were purchased from Sigma Chemical (St. Louis, MO).

### Cell viability assay

Inhibition of cell proliferation by chamaejasmine was measured by using MTT. Briefly, cells were subcultured in a 96-well plate with a density of 2 × 10^4^ cells/ml and incubated overnight at 37°C under an atmosphere of 95% O_2_ and 5% CO_2_. After 24 h, the medium was replaced with various concentration of chamaejasmine (0, 40, 80, and 160 μM). After 24- or 48-h incubation, culture media were modified for serum-free culture media. Then, the serum-free culture media containing MTT were discarded and DMSO was added to each well to dissolve the precipitate. The optical densities were measured at 490 nm spectral wavelength using Easy Reader 340 AT (SLT-Lab Instruments).

### Cell invasion assay

The effects of chamaejasmine on the MG-63 invasion were used in modified Boyden chambers consisting of transwell (Corning Costar Corp., U.S.A.). The upper surfaces of the transwell membranes were coated with 1 mg/ml Matrigel matrix (Collaborative Biomedical Products, U.S.A.) overnight. Cells were cultured in DMEM containing 40, 80, and 160 μM chamaejasmine and then plated into the top chambers. After incubation for 24 h at 37°C, cells on the upper surface of the membrane were removed with a cotton swab. The cells on the lower surface were stained with Crystal Violet for 20 min. Invading cells from three random fields were counted under a light microscope using a magnification of 200×.

### Cell apoptosis analysis by flow cytometry

The effect of chamaejasmine on cell apoptosis was analyzed by flow cytometer with Annexin V/PI kit (BD, Biosciences). Briefly, the medium was removed and the cells were treated with serum-reduced medium (0.5% FBS) with vehicle or different concentrations of chamaejasmine for 48 h. Cells were suspended in 300 μl Binding buffer, and then stained with Annexin-FITC and/or propium iodide. Positive controls for apoptosis were stained with only Annexin-FITC. Positive controls for necrosis were stained with only propium iodide. At least 10^4^ cells were analyzed by flow cytometer (BD, Biosciences).

### Caspase activity assays

Caspase activity assays in multi-well plate formats represent powerful tools for understanding experimental modulation of the apoptotic response. Caspase 3, Caspase 8, and Caspase 9 Multiplex Activity Assay Kit (Fluorometric) (ab219915) were purchased by Abcam. It a fluorescent assay that detects the activity of caspase 3/ 8/ 9 in cell lysates. We followed the protocol in https://www.abcam.com/ps/products/219/ab219915/documents/. Subtracting blank readings from all measurements (control and treated), using fluorescent intensity, determines fold change between control and treated cells. The fluorescence intensity was measured with FlexStation fluorescence microplate reader at the indicated wavelength. Caspase 3 (*E*x/*E*m = 535/620 nm), Caspase-8 (*E*x/*E*m = 490/525 nm), and caspase-9 (*E*x/*E*m = 370/450 nm), activities can be detected in a single assay without interferences from other caspases.

### Morphological apoptosis

Hoechst 33258 staining was carried out to observe morphological characteristics of apoptosis. In brief, cells were plated on the coverslips in 12-well plates and then treated with 0–160 μM chamaejasmine for 48 h. Then, the cells were washed twice with PBS, and then stained with Hoechst 33258 solution (5 μg/ml) for 10 min in the dark. Cells were observed using a fluorescence microscope (Olympus Optical Co., Japan) for morphological changes in the nucleus.

### Western blotting

MG-63 cells were lysed in RIPA buffer containing protease and phosphatase inhibitors. The protein concentrations were measured using a BCA protein assay kit (Beyotime, China). Proteins (40 mg) were separated by SDS-PAGE and transferred to a NC membrane (Bio-Rad, U.S.A.). After blocked with 5% non-fat milk for 1 h at room temperature, the membrane was incubated overnight at 4°C with antibodies against p-AMPK (1:1000), AMPK (1:1000), cleaved caspase 3 (1:1000), cleaved caspase 9 (1:1000), p-mTOR (1:1000), mTOR (1:1000), Bcl-2 (1:1000), Bax (1:1000), and β-actin (1:1000). After three washes, the membrane was incubated with secondary antibodies for 1 h at room temperature. The optical density of these protein bands was quantified using the Quantity One software after the separated proteins were visualized using ECL kits (Beyotime, China).

### Measurement of intracellular reactive oxygen species (ROS)

Intracellular ROS production was measured using a DCFH-DA fluorescent dye (Beyotime, China). MG-63 cells were cultured in six-well plates at a density of 1 × 10^5^/well. After different treatment, cells were incubated with 10 μM of DCFH-DA at 37°C for 15 min and then washed twice with ice PBS. Cells were observed using the fluorescence microscope mentioned above. Experiments were repeated at least three times to calculate the mean fluorescence intensity of DCFH-DA and data analyzed by Image Pro Plus.

### Lysotracker view

LysoTracker Green (Cell signaling technology, U.S.A.) were diluted by 50 μl DMSO and added into normal growth media for a working concentration of 50 nM. After specific treatment, cells were imaged live without fix by 4% paraformaldehyde. The labeled cells were observed at 200× magnifications under a fluorescence microscope.

### Transfection using siRNAs for gene silencing

Silence interfering (si) RNA oligonucleotides for AMPK was purchased from Cell signaling technology and transfected to suppress gene expression. A scrambled siRNA was used as a control. Transfection was performed with Lipofectamine 2000 (Invitrogen). According to the manufacturer’s directions, siRNA was mixed with Lipofectamine 2000 in serum-free media. The cells mixed with siRNA were diluted in complete medium and transfected for 6 h until changed the normal DMEM. Cells were cultured for another 48 h after transfection.

### Statistical analysis

All the experiments were performed in triplicate. Measurement data are expressed as mean ± SEM. Statistical analysis was performed with Graphpad Prism5. Statistical analyses were performed using the Student’s *t* test for comparisons of two groups and using one-way analysis of variance for multi-group comparisons. Significance was set at *P*<0.05.

## Results

### Chamaejasmine inhibits osteosarcoma cells viability

**Figure 1:**
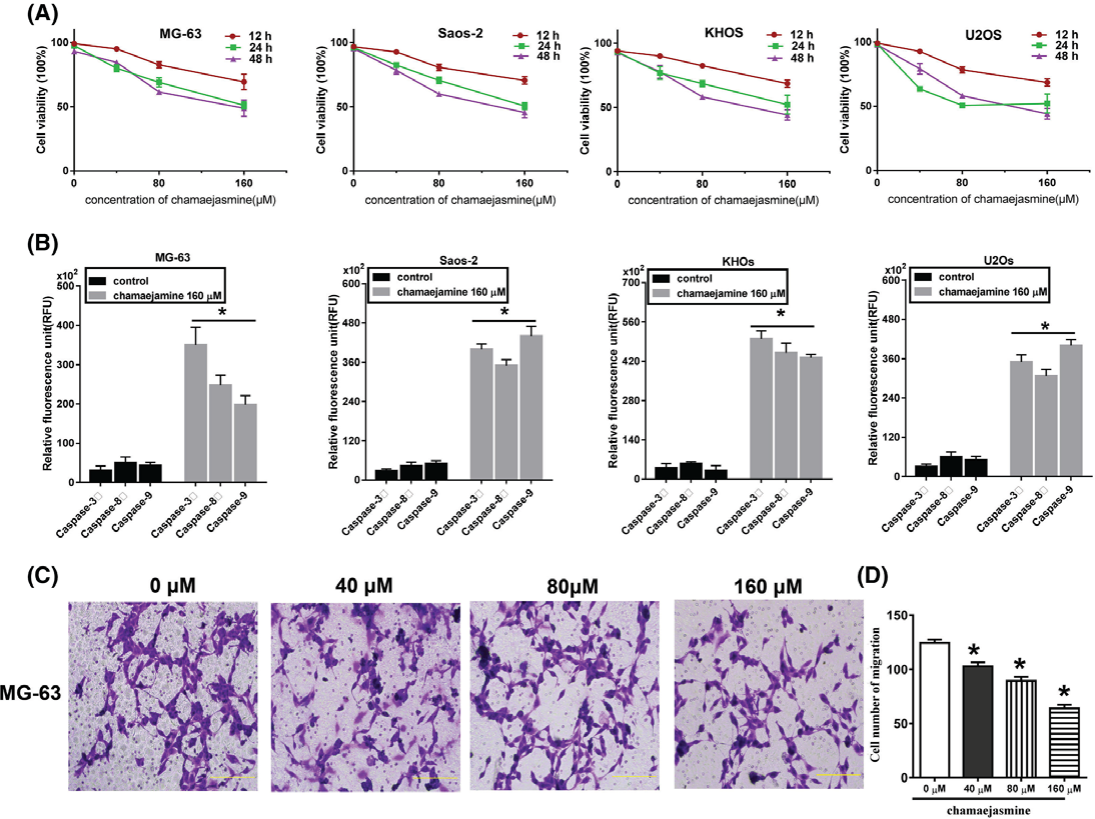
Chamaejasmine inhibits growth and induces apoptosis in OS cells (**A**) The viability of chamaejasmine in OS cells at three different time intervals and three different concentrations. Data are expressed as the mean ± S.E.M of at least three independent experiments. *P<0.05 vs 0 μM chamaejasmine. (**B**) Caspase activity of 3, 8 and 9 in MG-63, Saos-2, U2Os, and KHOs. The results were expressed as the mean ±S.E.M (*P<0.05 vs control group, *n*=3). (**C** and **D**) Cells with 0–160 µM chamaejasmine showed decreased invasive capacity compared with the control as documented by transwell invasion assay.

We assessed the effects of various doses of chamaejasmine on the viability of OS cells (MG-63, Saos-2, KHOS, and U2OS) using the MTT assay. As shown in Figure 1A, chamaejasmine inhibited the cell growth of cell lines effectively in a time- and dose-dependent manner (*n*=3, *P*<0.05). The growth inhibition caused by chamaejasmine is partially attributable to apoptosis, as evidenced by the activity of caspase; however, there are some differences among different cell line (Figure 1B). In MG-63 and KHOS cell lines, caspase-3 activity is significantly increased compared with control group. Whereas caspase-9 activity is much more sensitive and increasing in Saos-2 and U2OS cell lines. The invasion of the cells treated with chamaejasmine was significantly inhibited compared with that in the control cells by transwell invasion assay (Figure 1C,D). Our results revealed that the cytotoxic effect of the chamaejasmine showed a dose- and time-dependence.

### Chamaejasmine induces apoptosis in MG-63 cells

**Figure 2:**
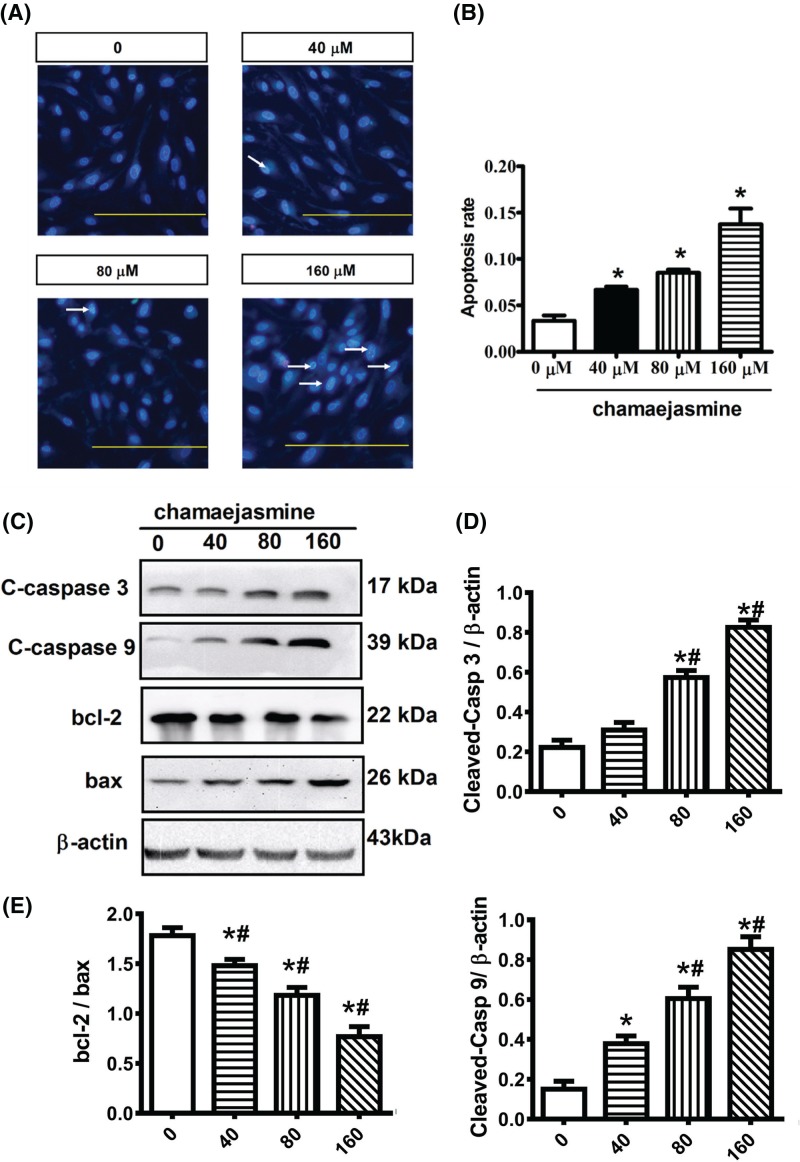
Effects of chamaejasmine on MG-63 cells apoptosis in concentration-dependent treatment groups (**A**) The number of apoptotic MG-63 cells from 0 to 160 µM chamaejasmine was assessed by Hoechst/PI staining and visualized at 200× magnification. (**B**) Data are the mean ± SEM (*n*=3). *P<0.05 compared with control group. (**C**) Proteins level of caspase 3, Bcl-2, Bax, and caspase- 3 determined by Western blotting assay. The upper trace of each group shows representative blots of the respective proteins, and the lower panels show the bar graphs summarizing the immunoblot data. (**D–F**) Densitometric results are expressed as a fold increase. Data are the mean ± SEM (*n*=3). *P<0.05 compared with control group; ^#^*P*<0.05 with 40 µM group.

We used Hoechst 33258 staining to investigate the apoptosis induced by chamaejasmine. As shown in Figure 2A, MG-63 cells were caused condensed and fragmented nuclei, as characteristic of apoptosis after treatment with different concentration of chamaejasmine for 48 h (Figure 2B). The protein expression of cleaved caspase 3 (c-caspase 3), cleaved caspase 9 (c-caspase 9), and bcl-2/bax were all remarkably increased after treatment with 160 μM chamaejasmine in MG-63 cells (Figure 2C). All the results indicate that chamaejasmine provokes cellular apoptosis by activating both the extrinsic and intrinsic pathways in dose-dependent manner.

### Chamaejasmine induces autophagy in MG-63 cells

**Figure 3:**
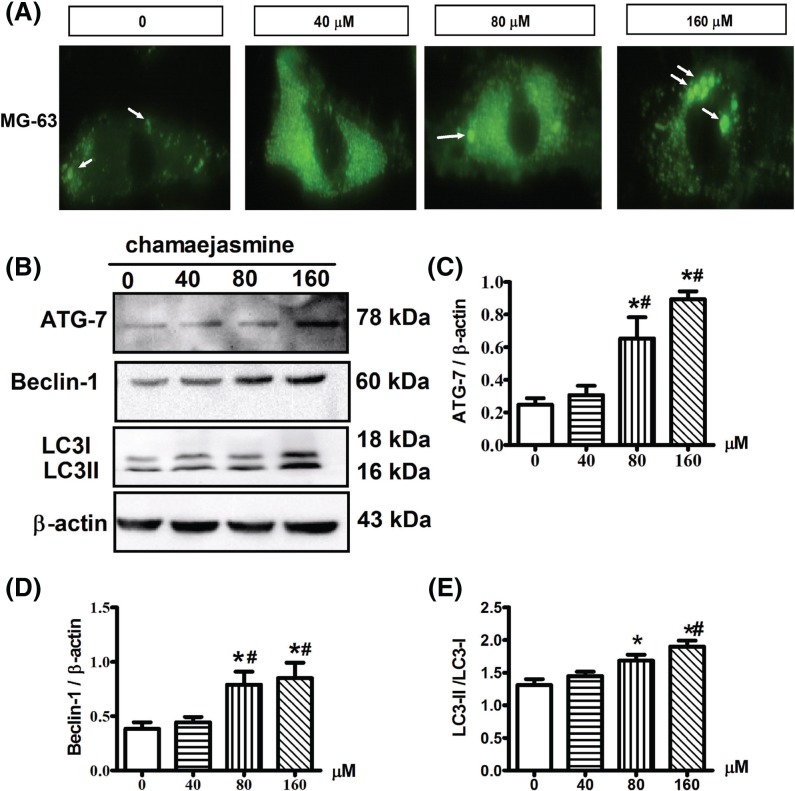
Protective autophagic effect of chamaejasmine on MG-63 cells (**A**) The punctate dots of a green fluorescent protein tagged of Lysotracker View in MG-63 cells after treatment with chamaejasmine 0–160 μM, magnification: 200×. (**B–E**) The ratio of LC3-II/I and the relative protein levels of ATG-7 and Becllin-1 was determined by densitometric analysis, and data were measured by Western Blot. β-Actin was used as an equal loading of proteins. Data are the mean ± SEM (*n*=3). *P<0.05 compared with control group; ^#^*P*<0.05, with 40 µM group.

We then determined whether chamaejasmine could induce autophagy in MG-63 cells. The increased acidic vesicular organelles (AVOs) are related to autophagosomes, which are the hallmark of autophagy [32]. We used LysoTracker Green to label lysosomes and autolysosomes. Our results showed that cells treated with chamaejasmine exhibited more AVOs in the cytoplasm (Figure 3A). Next we investigated the expression of autophagy-related proteins in cytoplasm by Western blotting analysis in chamaejasmine-treated and control cells. A significant conversion of LC3-I to LC3-II was detected after exposing MG-63 cells at 0 to 160 μM chamaejasmine for 48 h (Figure 3B). The accumulation of LC3-II positively correlated with the treatment dose, suggesting that chamaejasmine induced autophagy in a dose-dependent manner (Figure 3B). Our results demonstrated that chamaejasmine increased the level of ATG-7, LC3B-II and beclin-1 on MG-63 cells in concentration-dependent manner (Figure 3C–E).

### Chamaejasmine activates the activity of the AMPK/mTOR signaling pathway

**Figure 4:**
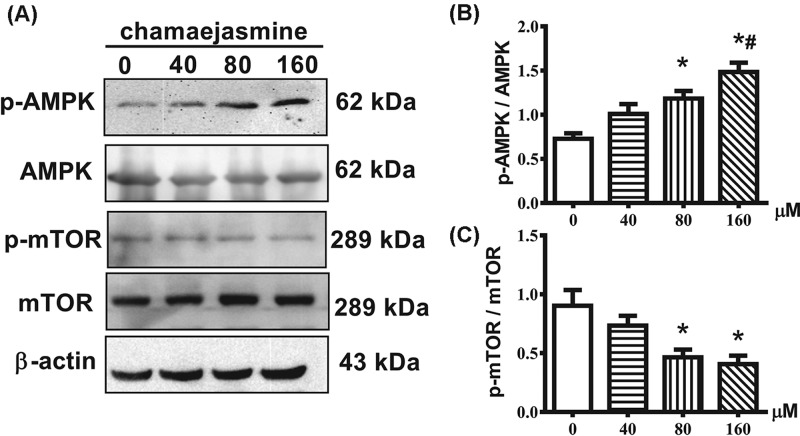
Chamaejasmine activates the AMPK/mTOR signaling pathway Total cell lysates were isolated 48 h after treatment with chamaejasmine of MG-63 cells and were subjected to immunoblot analysis with anti-pAMPK, -AMPK, p-mTOR, or –mTOR antibodies. β-Actin was used as a loading control. Results from three independent experiments are presented as means ± S.E.M. *P<0.05 compared with control group; ^#^*P*<0.05 with 40 µM group.

We investigated the effect of chamaejasmine on AMPK/mTOR signaling pathway to explore the molecular mechanism of autophagy activity. Our results showed that chamaejasmine increased the phosphorylation of AMPK and decreased the phosphorylation of mTOR in concentration-dependent manner (Figure 4).

### Chamaejasmine induced ROS production in MG-63 cells

**Figure 5:**
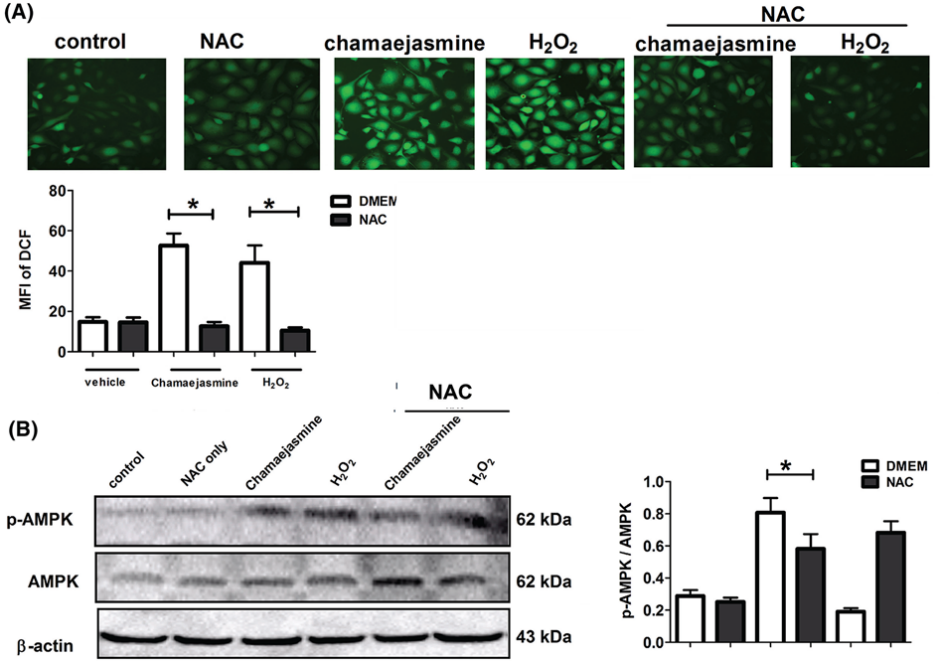
Effects of chamaejasmine on the ROS and p-AMPK expression in MG-63 cells (**A**) Random micrographs of dichlorofluorescein (DCF)-derived fluorescence in MG-63 cells from different treatment groups. The histogram showed the quantitative analysis of the mean fluorescence intensity in the indicated groups. Data are the mean ± SEM (*n*=3). DMEM only vs NAC treatment, *P<0.05. (**B**) Western blot of p-AMPK and AMPK proteins in MG-63 cells from different treatment groups. Data are the mean ± SEM (*n*=3). DMEM only vs NAC treatment, *P<0.05. DCF, dichlorodihydrofluorescein diacetate; ROS, reactive oxygen species; S.E.M, standard error of the mean.

To examine how chamaejasmine affects the production of ROS in MG-63 cells; DCFH-DA staining was performed. The production of ROS was increased in chamaejasmine-treated MG-63 cells compared with control group and NAC group (Figure 5A). Next, we investigated the underlying mechanism of AMPK activation by chamaejasmine in MG-63 cells. Significantly, the antioxidant NAC decreased the production of ROS and inhibited chamaejasmine-induced AMPK activation (Figure 5B). Meanwhile, hydrogen peroxide (H_2_O_2_) increased the ROS level and induced AMPK activation in MG-63 cells, prevented by NAC pre-administration. We conclude that chamaejasmine-induced AMPK activation requires ROS production.

### Inhibition of AMPK facilities chamaejasmine-induced apoptosis in MG-63cells

**Figure 6:**
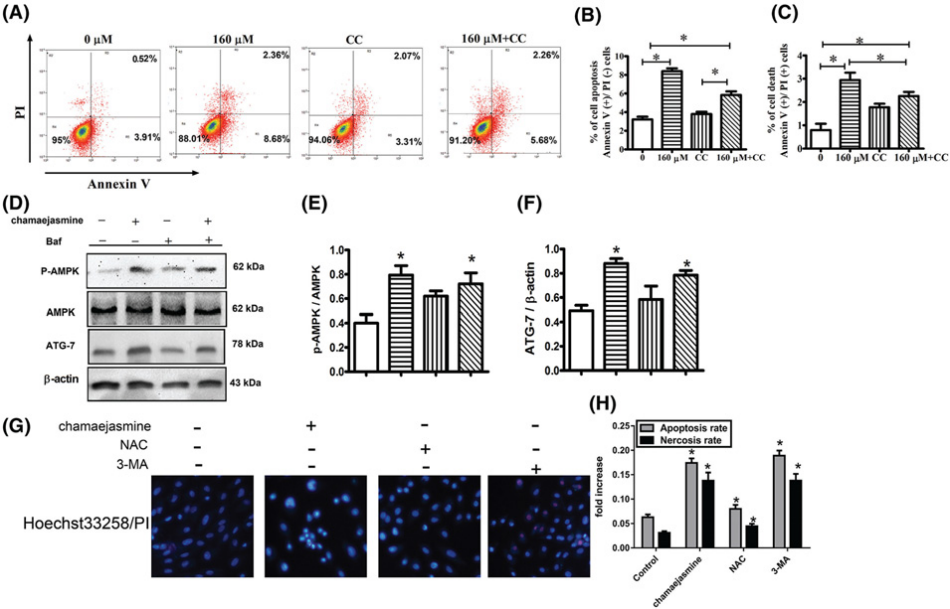
Activation of AMPK mediates chamaejasmine-induced autophagy in osteoblastoma cells (**A–C**) The cells were treated with 0 µM (control), 160 µM chamaejasmine, CC and CC+160 µM chamaejasmine for 48 h and analyzed using fluorescence-activated cell sorting. R1, R2, R3, and R4 quadrants show percentage of necrotic, early apoptotic, normal healthy, and late apoptotic cell populations, respectively. CC, Compound C; 160 µM, chamaejasmine; PI, propidium iodide. (**D–F**) MG-63 cells were divided into control, or 160 μM of chamaejasmine, and/or with Baf treatment group. Phosphor (p)- and total AMPK and ATG-7 were examined by Western blotting and quantified by Image Lab. Data were summarized from at least three different experiments (*P < 0.05 vs control). (**G–H**) MG-63 cells were treated by chamaejasmine and NAC with 3-MA. Representative photographs of double staining of PI and Hoechst 33258. The apoptotic cells were observed as nuclei pyknosis by Hoechst 33258. PI positive cells (red/pink) are regarded as the necrotic cells. The results were expressed as the mean ± S.E.M (*P<0.05 vs control group, *n*=3).

To investigate whether chamaejasmine-induced apoptosis and autophagy have a connection with AMPK/mTOR signaling pathway, we used bafilomycin-A1 (Baf) (autophagy inhibitor, 100 nM) and Compound C (AMPK inhibitor, 2.5 μM) for further exploration. When the cells were treated with Compound C for 48 h, the proportion of Annexin V-staining positive cells (total apoptotic cells) markedly increased from 4.43% in control to 5.38%, respectively. Thus, flow cytometric analysis demonstrated that Compound C could reverse the cell apoptosis induced by chamaejasmine (Figure 6A). Chamaejasmine obviously increased the activity of p-AMPK and ATG-7 in MG-63 cells, while Baf (100 nM) decreased the level of p-ampk/ampk and the expression of ATG-7. In addition, the Baf markedly decreased the effects of chamaejasmine in the chamaejasmine +Baf co-treatment group. It proved that the AMPK pathway signaling was involved in chamaejasmine-mediated autophagy role in MG-63 cells (Figure 6D–F). In order to demonstrate the effect of chamaejasmine on the relationship between apoptosis and autophagy, MG-63 cells were treated with 3-MA, an autophagy inhibitor. Our data showed that cell apoptosis ratio was increased in MG-63 cells after treatment with chamaejasmine **or** 3-MA (Figure 6G,H) compared with chamaejasmine group. Whereas there was no significant changes between chamaejasmine and 3-MA groups. Our result revealed that apoptosis was possible partly dependent on autophagy.

### AMPK silencing impairs chamaejasmine-induced cell apoptosis and autophagy

**Figure 7:**
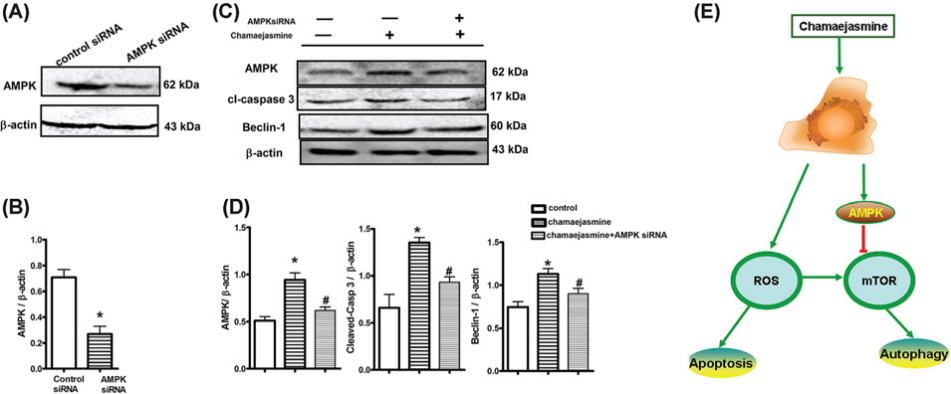
Effects of AMPK-siRNA on chamaejasmine-elicited apoptosis (**A** and **B**) Suppression of autophagy in MG-63 cells were transfected with AMPK siRNA at 150 nM for 72 h. Protein levels of AMPK were measured by Western blotting with anti-AMPK antibody. (**C** and **D**) MG-63 cells were transfected with control siRNA or with AMPK siRNAs. Cells with AMPK siRNA transfection were co-incubated with chamaejasmine for 48 h. The upper trace of each group shows representative blots of the respective proteins, and the lower panels show the bar graphs summarizing the immunoblot data. Densitometry results are expressed as a fold increase. *P<0.05 vs control siRNA group; #*P*<0.05 vs chamaejasmine group. (**E**) Schematic diagram of the mechanisms of chamaejasmine-dependent apoptosis and autophagy in regulation of ROS and AMPK in osteosarcoma cells. Chamaejasmine-induced autophagy elicited cytotoxic effects through activated-AMPK-mTOR pathway and suggested a novel mechanism of chamaejasmine-induced MG-63 cells inhibited effect.

To assess the effect of AMPK inhibition on signal transduction rather than a modulation of other cellular activities, accounting for the chamaejasmine-induced cell apoptosis and autophagy in MG-63 cells, we analyzed cleaved caspase 3 and Beclin-1 after silence interfering RNA (siRNA)-mediated silencing of AMPK. As shown in Figure 7A,B, the target-specific siRNA of AMPK significantly suppressed the cellular AMPK level by about 70% in chamaejasmine-treated MG-63 cells. Cells with AMPK knockdown led to a large decrease of cleaved caspase 3. AMPK knockdown also inhibited Beclin-1 after chamaejasmine treatment (Figure 7C,D). These results indicate that activation of AMPK is partly responsible for the increased cellular apoptosis and autophagy. All above findings suggest that apoptosis and autophagy induced by chamaejasmine in MG-63 cells could be mediated by the activation of AMPK/mTOR signaling pathways.

## Discussion

OS, the most common malignant bone tumor, occurs predominantly in children and adolescents. Until recently, a 5-year survival rate has been improved through surgery combined with adjuvant and neoadjuvant chemotherapy and radiotherapy [33,34]. However, recurrence, metastasis, and drug resistance are responsible for the poor effectiveness of cytotoxic drugs [35]. Therefore, it was urgent to develop novel treatments with potent anti-tumor effects to target the malignant behavior of OS cells and improving the treatments for patients.

In the past few years, researchers have reported that chamaejasmine can induce apoptosis in multiple cancer cell lines [36–38]. However, the exactly mechanism of the cause for apoptosis or autophagy of tumor cells remains unclear. We have demonstrated that chamaejasmine could induce apoptosis via p53 pathway in osteosarcoma cells [38]. ROS at a low concentration contribute to the improvement of cellular function and cell survival whereas excessive ROS causes the capability to promote programmed cell death [39]. We hypothesized that caspase and AMPK/mTOR signaling pathway may take part in this process. Our study first revealed that chamaejasmine-induced apoptosis and inhibited growth of MG-63 cells via AMPK activation. Here we report that chamaejasmine-induced early autophagy is mainly dependent on the induction of autophagosomes, conversion of LC3-I to LC3-II, expression of Atg7 and Beclin-1 and inhibition of Bcl-2/Bax, caspase 3, and caspase 9.

In the present study, after treatment with different chamaejasmine concentrations (0–160 μM) for 24 and 48 h, MTT assays showed that the cell viability was significantly decreased in a concentration- and time-dependent manner in MG-63 cells (Figure 1A). The effect of chamaejasmine on cell invasion, hallmark of malignancy, was examined by transwell assay. Our study demonstrated that chamaejasmine reduced cell invasion in a concentration-dependent manner (Figure 1C).

Apoptosis is a key strategy for protective mechanism against tumor biogenesis. Many anti-cancer drugs play roles in apoptosis induction and cell arrest eradicating the cancerous cells [40]. It has been identified that there are two distinct pathways of apoptosis in mammalian cells: the extrinsic and intrinsic pathways [41]. The extrinsic pathway is activated by pro-apoptotic receptor signals at the cellular surface; the intrinsic pathway is governed by Bcl-2-family proteins members, which affect the release of cytochrome *C* into the cytosol, resulting in caspase 9 and 3 activation [42,43]. The apoptosis induced by chamaejasmine was further confirmed in a concentration-dependent manner by Hoechst staining fluorescence imaging (Figure 2A). Our study demonstrated a decrease in the ratio of Bcl-2/Bax in MG-63 cells after treatment with different concentrations of chamaejasmine. Meanwhile, chamaejasmine-induced apoptosis was mediated by caspase 9 and caspase 3 in MG-63 cells (Figure 2C-F).

It has been mentioned that AMPK activation is involved in cell growth and reprogramming metabolism and autophagy through regulating its many downstream kinases [44,45]. Because AMPK plays a critical role in response to autophagy [27], we assessed the effect of chamaejasmine on AMPK pathway in osteosarcoma. It remains controversial about how autophagy modulates the balance between cytoprotection and cell death through AMPK pathway. Existing research demonstrated that activation of AMPK might inhibit cell growth and induce cancer cell apoptosis under stress condition [20,45]. While other studies indicate that AMPK is pro-survival and anti-apoptotic [46]. In addition, previous reports have established p-AMPK/mTOR serving as a key signaling pathway, which negatively regulates apoptosis and autophagy [47] in glucose/glycogen metabolism. ROS is well-known as the activator of AMPK [48,49] and directly induces autophagy by up-regulating autophagy-associated gene (ATG) expression [50]. The mechanism of chamaejasmine-mediated induction of oxidative stress is not clear. Here, we have provided evidence to support that ROS production and cancer cell apoptosis are involved in AMPK activation by chamaejasmine. In our study, ROS and AMPK activation significantly increased after chamaejasmine treatment (Figure 5). The AMPK inhibitor, Compound C, significantly inhibited the induction of apoptosis by chamaejasmine (Figure 6A). Indeed, while an increase in LC3B-II level in steady state conditions corresponds to an increase in the amount of autophagosomes in cells (Figure 3B), this may be due to activation or late inhibition of the autophagic process. Therefore, in order to distinguish between these opposite circumstances, it is necessary to compare autophagic-related proteins with those of the corresponding samples treated with lysosomal protease inhibitors (such as Bafilomycin A1 and Chloroquine): if autophagic flux is increased, the amount of LC3B-II or ATG-7 or Beclin-1 will be higher in presence of inhibitors (the autophagic process is active) while, if the autophagic process is inhibited, the amount of LC3B-II or ATG-7 or Beclin-1 will not increase in presence of inhibitors (the flux is blocked). Through exploring the further mechanism signaling of AMPK, NAC also decreased chamaejasmine-induced AMPK activation, suggesting that ROS production might be required for AMPK activation and cell autophagy by chamaejasmine. As a matter of fact, AMPK activation by chamaejasmine could activate oxidative stress and to increase the apoptotic cells.

Therefore, we investigated the relationships between chamaejasmine-induced apoptosis and autophagy. Autophagy and apoptosis control the turnover of organelles and proteins within cells, and of cells within organisms, respectively, and many stress pathways sequentially elicit autophagy, and apoptosis within the same cell. Generally, autophagy blocks the induction of apoptosis, and apoptosis-associated caspase activation shuts off the autophagic process. However, in some cases, autophagy or autophagy-relevant proteins may help to induce apoptosis or necrosis. In our study, co-incubation chamaejasmine with Baf also increased the expressions of p-AMPK/AMPK and ATG-7 (Figure 6D), indicating that the autophagy induced by chamaejasmine via the activation of AMPK. In order to demonstrate the effect of chamaejasmine on the relationship between apoptosis and autophagy, the administration of 3-MA, an autophagy inhibitor, treated with MG-63 cells. Our data showed that the increasing cell apoptosis ratio in MG-63 cells treated with chamaejasmine (Supplement 1), NAC, and 3-MA decreased compared with chamaejasmine group in MG-63 cells. Our result revealed that apoptosis was possible partly dependent on autophagy.

The important role in regulation apoptosis and autophagy is the cellular redox state. Activation of AMPK could influence the balance between the formation and degradation of intracellular ROS, and further influence the redox state of cell [51]. Therefore, the relationship between autophagy and apoptosis induced by chamaejasmine might help regulate these processes and improve the anti-tumor effects of chamaejasmine. AMPK inhibition by Compound C or AMPKsiRNA was added for the mechanism study. Compound C as an AMPK inhibitor decreases AMPK expression. However, Compound C may also regulate the expression of other proteins, such as AKTT308, AKTS473 or CK1, CAMMK according to previous studies. Therefore, we used AMPKsiRNA to demonstrate the phenotypic alteration by biological inhibition of AMPK. As shown in Figure 7C, AMPK inhibition decreased the expression of cleaved-caspase 3 in MG-63 cells; however, we could not figure out the exactly mechanism of chamaejasmin in the relationship between apoptosis and autophagy. More interestingly, as designed in our experiments, there were not any physical contact between chamaejasmine and MG-63 cells, which means there must be one or some secretory protein(s) that bridged the communication between cells and triggered autophagy. Identification of such soluble mediators is needed and will be essential for depicting the detailed molecular behaviors of chamaejasmine in the cross-talk between autophagy and apoptosis. As depicted in Figure 7E, chamaejasmine appears to regulate process and program that might affect the growth and/or survival of cancer cells.

## Conclusion

Our findings indicated that chamaejasmine exerted its cytotoxic effect against MG-63 cells through the activation of AMPK/mTOR pathway, which was responsible for autophagy. However, further study is needed to explore whether chamaejasmine is suitable for providing an attractive target for cancer therapy. For example, further analysis is required to investigate its cytotoxic potential *in vivo* models as a promising anti-cancer medicine for osteosarcoma therapy. In addition to assessing the possibility of chamaejasmine, more information is needed to determine whether AMPK pathway contributes to the association with apoptosis and autophagy during osteosarcoma.

## Data Availability

The datasets used and/or analyzed during the current study are available from the corresponding author on reasonable request.

## Supporting Data Availability

We declared that materials described in the manuscript, including all relevant raw data, will be freely available to any scientist wishing to use them for non-commercial purposes, without breaching participant confidentiality.

## Abbreviations

3-MA: 3-methyladenine
AMPK: adenosine 5′-monophosphate (AMP)-activated protein kinase
Baf: bafilomycin-A1
CC: Compound C
mTOR: mammalian target of rapamycin
NAC: N-acetyl-L-cysteine
ROS: reactive oxygen species
